# Perceived Benefits and Barriers of Breast Self‐Examination and Mammography for Breast Cancer Screening in the PERSIAN Guilan Cohort Study (PGCS) Population

**DOI:** 10.1002/cnr2.70459

**Published:** 2026-01-29

**Authors:** Farahnaz Joukar, Sara Zakaryapour, Fateme Sheida, Faezeh Fashkhami, Zahra Atrkar‐Roshan, Farideh Hasavari, Fariborz Mansour‐Ghanaei

**Affiliations:** ^1^ Gastrointestinal and Liver Diseases Research Center Guilan University of Medical Sciences Rasht Iran; ^2^ Department of Nursing, School of Nursing and Midwifery Guilan University of Medical Sciences Rasht Iran; ^3^ Guilan University of Medical Sciences Rasht Iran; ^4^ Department of Biostatistics, School of Medicine Guilan University of Medical Sciences Rasht Iran

**Keywords:** breast cancer, cohort study, mammography, screening

## Abstract

**Background:**

Breast examination and mammography help in the detection of breast cancer and are valid in improving survival by reducing mortality.

**Aims:**

In this study, we aimed to investigate women's knowledge of breast cancer screening in the Prospective Epidemiological Research Studies (PERSIAN) Guilan Cohort Study (PGCS) population.

**Methods and Results:**

This cross‐sectional study was conducted on 476 women aged 35–70 among the PGCS population. The demographic and clinical data of participants were collected through a questionnaire. Also, the Champion Health Belief Model Scale, including the perceived benefits of breast self‐examination (six phrases), perceived barriers to breast self‐examination (nine phrases), perceived benefits of mammography (six phrases), and perceived barriers to mammography (nine phrases), was used to collect the knowledge data. The variables of the questionnaire were assessed using the Likert scale. Data were analyzed using SPSS version 20 by significant level < 0.05.

**Results:**

Most of the research subjects were within the age of 45–55 years (35.9%) and most of them (64.9%) did not mention any history of prior mammography, but among those with positive history of mammography, most of them (55.1%) had done it without any problem and only based on recommendation. The average scores of benefits and barriers for breast self‐examination among the participants were 9.9 ± 3.7 and 15.2 ± 4.6, respectively. Similarly, for mammography, the average scores were 9.9 ± 3.6 and 14.2 ± 4.7. In overall, factors including age 35–45 years, having insurance, higher education levels, having former visit of a doctor due to breast problem, family history of breast cancer in first degree relatives, and positive history of performing mammography were associated with better scores of perceived benefits and barriers in both breast self‐examination and mammography (*p* ≤ 0.05).

**Conclusion:**

According to the barriers and benefits identified in this study, it is possible and also necessary to plan for breast cancer screening with organized regional educational plans. It is recommended to focus more on attracting older women to perform screening programs. It is also necessary to encourage doctors to refer women for mammography and support insurance organizations to provide screening services at a lower cost.

## 1 Introduction

Breast cancer is the leading cause of cancer‐related deaths among women worldwide, with a significant increase in diagnoses over the past 5 years, affecting 7.8 million women. According to 2020 statistics, there were 2.3 million breast cancer new cases and 685 000 related deaths, globally [1]. Breast cancer incidence demonstrates approximately a fourfold variation across World Health Organization (WHO) regions. The highest incidence rates (per 100 000 women) are reported in Australia/New Zealand (95.5), Western Europe (90.7), and Northern America (89.4), whereas the lowest rates are observed in South‐Central Asia (26.2), Middle and Eastern Africa (33.0), and Central America (39.5) [2]. A recent meta‐analysis of 25 studies in Iran as a low‐ and middle‐income, developing nation in the Middle East, revealed a pooled age‐standardized incidence rate (ASIR) of breast cancer of 32 per 100 000 women [3]. With considering the higher mortality rates in developing versus developed countries, these substantial disparities in breast cancer incidence may, in part, be attributed to differences in the availability and implementation of population‐based screening programs. Developed countries have successfully reduced mortality rates through early detection, improved access to diagnostic and treatment facilities, and the adoption of effective health practices and behaviors, including screening [2, 4].

Breast cancer can be identified at an early stage through appropriate screening methods, leading to more successful treatment outcomes. Therefore, participation in diagnostic and screening programs is crucial [5, 6]. The Ministries of Health and global guidelines advise women aged 40 and above to undergo annual mammograms [7, 8]. Mammography is an effective tool for early cancer detection and reducing mortality rates, as it can detect breast tumors 1–3 years before they can be felt [9, 10]. Despite the benefits of screening in reducing breast cancer incidence, a small proportion of women undergo screening [11]. Factors such as social norms, cultural sensitivity, socioeconomic status, lack of awareness, psychological disorders, fear of cancer diagnosis, and negative attitudes toward the results influence women's participation in early detection programs [11, 12, 13]. Studies conducted in Iran have identified barriers such as poor doctor‐patient interactions, gender mismatch with healthcare providers, anxiety and fear related to cancer diagnosis, lack of knowledge about breast cancer, long distances to screening centers, and lack of insurance coverage as obstacles to breast cancer screening [14, 15]. To effectively address these barriers and reduce disparities, it is crucial to build a structured and in‐depth understanding of the specific challenges that different populations face in accessing breast cancer screening. Gaining this insight allows for the development of targeted, evidence‐informed strategies that are not only culturally appropriate but also make the best use of available resources [16].

To the best of our knowledge, no previous cohort‐based study has been conducted in Guilan Province, northern Iran, to examine women's perceived benefits and barriers of breast cancer screening, particularly through the Champion Health Belief Model Scale, a widely validated tool for understanding health behaviors. This gap in the literature highlights a critical need for region‐specific data to inform effective screening interventions. Therefore, our study aims to evaluate these perceptions among women in the Prospective Epidemiological Research Studies (PERSIAN) Guilan Cohort study (PGCS) population.

## 2 Methods

### 2.1 Study Design and Subjects

This cross‐sectional‐analytical study is a part of the PGCS with a 10 520 population in Guilan province, Iran [17, 18]. Sample size was calculated based on Fouladi et al. study [19] and with formula *n* = $\frac{Z^{2}{\mathrm{SD}}^{2}}{d^{2}}$, considering *α* = 0.05, *Z* = 1.96, SD = 8.91, and *d* = 0.8, which yielded an estimated sample size of *n* = 476. All women from the PGCS population with no previous history of breast cancer were considered eligible for the study; however, individuals who declined to participate were excluded. Finally, among 5634 women in PGCS population, 476 individuals were included for the study by systematic random sampling method based on the mentioned criteria. The study was approved by the ethical committee of the Guilan University of Medical Sciences, Rasht, Iran (IR.GUMS.REC.1397.363).

### 2.2 Data Collection and Questionnaire

The demographic and clinical data were age, marital status, employment status, health insurance, place of residence, education, history of breast cancer screening, personal history of any benign breast lesions, presence of breast problems or discomfort, type of breast problem, history of first‐degree relatives with breast cancer, history of infertility, history of miscarriage, and history of pregnancy. The Champion Health Belief Model Scale, a self‐report instrument firstly constructed in 1993 and revised in 1997 and 1999, was used to investigate breast cancer screening behaviors, including the benefits and barriers of breast cancer screening [20, 21, 22]. This questionnaire was validated, and its reliability was assessed in Iran by Taymoori and Berry [23] with a Cronbach's *α* coefficient of 86% for the Persian version. The Champion Health Belief Model Scale used in the present study includes the following items: the perceived benefits of breast self‐examination (BSE) (six phrases), perceived barriers to BSE (nine phrases), perceived benefits of mammography (six phrases), and perceived barriers of mammography (nine phrases).

The variables of the questionnaire were evaluated using a 5‐point Likert scale for the benefit statements (*strongly disagree* (1), *disagree* (2), *neutral* (3), *agree* (4), *strongly agree* (5)) and for the barrier statements (*strongly disagree* (5), *disagree* (4), *neutral* (3), *agree* (2), *strongly agree* (1)). After completing the sampling, the strongly agree and agree statements were merged and analyzed as agreement, while the strongly disagree and disagree statements were merged and analyzed as disagreement. Therefore, the benefit statements were analyzed as (*disagree* (1), *neutral* (0), *agree* (2)), and the barrier statements were analyzed as (*disagree* (2), *neutral* (0), *agree* (1)). For both BSE and Mammography, the range of scores for benefits and barriers was 6–12 and 9–18, respectively. All data were collected through face‐to‐face interview.

### 2.3 Statistical Analysis

The data was reported as percentages, numbers, and mean ± standard deviation (SD). An independent *t*‐test was used to compare variables among participants. Multivariate regression was applied to evaluate the association between predictive factors with perceived benefits and barriers for BSE and mammography. The data was analyzed using SPSS version 20, and the significance level was considered to be less than 0.05.

## 3 Results

This cross‐sectional study was performed on a sample of 476 women, aged 35–70, from the PGCS population. The sociodemographic analysis of the participants showed that the majority were within the age of 45–55 years (35.9%), were married (85.7%), identified as housewives (73%), had health insurance (84.5%), resided in urban areas (66.4%), and just 16.8% of them had completed a diploma or university education. Around 35% of the participants in the study (*n* = 167) had done a prior mammography. Most of the research subjects (64.9%) did not mention any history of prior mammography, but among those with positive history of mammography, most of them (55.1%) had done it without any problem and only based on recommendation. 83% of the participants reported negative previous history of breast abnormalities or discomfort, while the remaining individuals reported experiencing these issues at least once. Only 15.5% of those who had a prior history of breast issues received medical care, with the most common complication being the experience of breast pain (7.7%). The majority of the research participants (96.2%) did not have any cases of breast cancer among their first degree relatives, as well as reporting no history of infertility (87.2%) or stillbirth (94.7%). 71.4% of the participants reported a history of one abortion and most of them had a history of pregnancy (93.9%) (Table 1).

**TABLE 1:** Demographical and clinical data of the participants.

Variable			Frequency, *n* (%)
Age (years)	35–45		151 (31.7)
	45–55		171 (35.9)
	55–65		119 (25.0)
	≥ 65		35 (7.4)
Marital status	Single		17 (3.6)
	Married		408 (85.7)
	Widow		44 (9.2)
	Divorced		7 (1.5)
Occupation	Employee		129 (27.1)
	House wife		347 (72.9)
Insurance	Yes		402 (84.5)
	No		74 (15.5)
Habitat	Urban		316 (66.4)
	Rural		160 (33.6)
Education	Illiterate		2 (19.3)
	Under diploma		304 (63.9)
	Diploma and higher		80 (16.8)
Having any prior problem in the breast	Yes	Visit a doctor	74 (15.5)
		Failure to visit a doctor	7 (1.5)
	No		39 (83.0)
Types of breast problem	Breast pain		37 (7.7)
	Benign breast diseases		32 (6.8)
	Discharge, infection, and abscess		12 (2.6)
	None		395 (82.9)
Breast cancer in first degree relatives	Yes		18 (3.8)
	No		458 (96.2)
History of infertility	Yes		61 (12.8)
	No		415 (87.2)
History of stillbirth	Yes		25 (5.3)
	No		451 (94.7)
Number of abortions	Once		126 (71.4)
	Twice		42 (24.0)
	More than twice		8 (4.6)
History of pregnancy	Yes		447 (93.9)
	No		29 (6.1)
History of mammography	Yes		167 (35.1)
	No		309 (64.9)
Reason for mammography	Pain or mass in the breast		36 (21.6)
	Without breast problems and with the doctor's advice		39 (23.4)
	Without breast problems and with the advice of others		92 (55.1)

The rate of responses related to each component of the Champion Health Belief Model Scale assessing the perceived benefits and barriers of BSE and mammography is presented in Table 2. In detail, according to the results related to the perceived benefits of BSE (Table 2), 436 women (91.6%) believed that monthly BSE aids in identifying breast lumps earlier, and 410 cases (86.1%) agreed that BSE may reduce the likelihood of dying from breast cancer. The most frequent perceived barrier of BSE was lack of the ability to do breast examination correctly (41.6%). Most participants agreed with the benefits of mammography (ranging from 70.6%–86.1% of agreement for various statements) and the most frequent perceived benefit was “Having a mammogram will help me find breast lumps early.” In terms of perceived barriers of mammography, the most frequent item was “Having a mammogram takes too much time,” in about 33% of the participants (Table 2).

**TABLE 2:** The type of responses of perceived benefits and barriers for (a) BSE and (b) mammography.

Questionnaire item		Agree	No idea	Disagree
*(a) Perceived benefits and barriers of BSE*				
Benefits	When I do self‐examination, I feel self‐satisfied.	381 (80)	94 (19.7)	1 (0.2)
	When I complete monthly BSE I don't worry as much about breast cancer.	408 (85.7)	64 (13.4)	4 (0.8)
	Completing BSE each month will allow me to find lumps early.	436 (91.6)	40 (8.4)	0 (0)
	Completing BSE each month may decrease my chances of dying of breast cancer.	410 (86.1)	66 (13.9)	0 (0)
	Completing BSE each month will decrease requiring radical or disfiguring surgery if breast cancer occurs.	344 (72.3)	132 (27.7)	0 (0)
	Completing BSE each month may help me find breast lumps early before it is detected by a doctor.	349 (81.7)	86 (18.1)	1 (0.2)
Barriers	I don't feel I can do breast examination correctly.	198 (41.6)	29 (6.1)	249 (52.3)
	Doing breast examination will make me worry about what is wrong with my breast.	20 (4.2)	35 (7.4)	421 (88.4)
	BSE is embarrassing to me.	5 (1.1)	32 (6.7)	439 (92.2)
	BSE takes too much time.	64 (13.4)	39 (8.2)	373 (78.4)
	It is hard to remember to do breast examination	57 (12)	39 (8.2)	380 (79.8)
	I don't have enough privacy to do breast examination.	0 (0)	33 (6.9)	443 (93.1)
	BSE is not necessary if you have a breast examination by a healthcare provider.	35 (7.4)	98 (20.6)	343 (72.1)
	BSE is not necessary if you have a routine mammogram.	25 (5.3)	129 (27.1)	322 (67.6)
	My breasts are too lumpy for me to complete breast examination.	2 (0.4)	35 (7.4)	439 (92.2)
*(b) Perceived benefits and barriers of mammography*				
Benefits	When I get a recommended mammogram, I feel self‐satisfied.	336 (70.6)	137 (28.8)	3 (0.6)
	If I get a mammogram and nothing is found, I don't worry as much about breast cancer.	398 (83.6)	71 (14.9)	7 (1.5)
	Having a mammogram will help me find breast lumps early.	424 (89.1)	52 (10.9)	0 (0.0)
	Having mammogram will decrease my chances of dying from breast cancer.	399 (83.8)	77 (16.2)	0 (0.0)
	If I find a lump through a mammogram, my treatment for breast cancer may not be as bad.	391 (82.1)	85 (17.9)	0 (0.0)
	Having a mammogram is the best way for me to find a very small lump.	420 (88.2)	54 (11.3)	2 (0.4)
Barriers	I am afraid to have a mammogram because I might find out something is wrong.	60 (12.6)	37 (7.8)	379 (79.6)
	I am afraid to have a mammogram because I don't understand what will be done.	60 (12.6)	37 (7.8)	379 (79.6)
	I don't know how to go about getting a mammogram.	116 (24.4)	37 (7.8)	323 (67.9)
	Having a mammogram is too embarrassing.	8 (1.7)	36 (7.6)	432 (90.8)
	Having a mammogram takes too much time.	159 (33.4)	50 (10.)	267 (56.1)
	Having a mammogram is too painful.	32 (6.7)	158 (33.2)	286 (60.1)
	I cannot remember to schedule a mammogram.	121 (25.4)	56 (11.8)	299 (62.8)
	I have other problems more important than getting a mammogram.	266 (5.9)	35 (7.4)	175 (36.8)
	I am too old to need a routine mammogram.	18 (3.8)	36 (7.6)	422 (88.7)

Abbreviation: BSE: breast self‐examination.

The average scores of benefits and berries for BSE among the participants were 9.9 ± 3.7 and 15.2 ± 4.6, respectively. Similarly, for mammography, the average scores were 9.9 ± 3.6 and 14.2 ± 4.7. The average scores according to all demographic and clinical characteristics are shown in Table 3. In this study, in overall, factors including age 35–45 years, having insurance, higher education levels, having former visit of a doctor due to breast problem, family history of breast cancer in first degree relatives, and positive history of performing mammography were associated with better scores of perceived benefits and barriers in both BSE and mammography (*p* ≤ 0.05). On the other hand, elderly people (> 65 years), farmers and ranchers, having no insurance, being illiterate, no history of breast cancer in first degree relatives and no history of mammography, significantly were associated with less perceived benefits of both BSE and mammography (*p* > 0.05).

**TABLE 3:** The average scores of perceived benefits and barriers for BSE and mammography and associated factors.

Variable			Mammography				BSE			
			Score of benefits, mean ± SD	*p*	Score of barriers, mean ± SD	*p*	Score of benefits, mean ± SD	*p*	Score of barriers, mean ± SD	*p*
Age (years)	35–45		10.8 ± 2.3	**0.001**	15.4 ± 3.1	**0.001**	10.5 ± 2.9	**0.030**	15.9 ± 3.4	**0.010**
	45–55		9.6 ± 4		13.8 ± 5.1		10.1 ± 3.7		15.1 ± 4.9	
	55–65		9.7 ± 4		13.9 ± 5.1		9.5 ± 4.1		14.8 ± 4.9	
	≥ 65		8.6 ± 4.6		12.5 ± 6.1		8.7 ± 4.6		13.1 ± 6.1	
Marital status	Single		7.6 ± 4.5	**0.005**	12 ± 5.9	0.200	9.1 ± 4.6	0.300	13.7 ± 6.8	0.300
	Married		10.1 ± 3.6		14.3 ± 4.6		9.9 ± 3.7		15.2 ± 4.5	
	Widow		10.4 ± 0.9		14.6 ± 4.1		9.8 ± 3.8		14.9 ± 4.7	
	Divorced		9.8 ± 4.4		13.6 ± 6.3		9.8 ± 3.4		17.4 ± 0.8	
Occupation	Employee		10.1 ± 4.8	**0.001**	15.7 ± 2.7	**0.020**	10.0 ± 4.8	**0.010**	15.8 ± 3.4	0.040
	Self‐employment		11.3 ± 1.1		16.5 ± 1.7		11.3 ± 1.3		16.2 ± 2.7	
	House wife		10.3 ± 3.3		14.5 ± 4.3		10.2 ± 3.4		15.5 ± 4.2	
	Retired		11.3 ± 1.5		14.8 ± 1.8		11.2 ± 1.4		16.5 ± 2.8	
	Farmer/rancher		8.5 ± 4.6		13.8 ± 5.9		8.8 ± 4.5		16.8 ± 5.8	
Insurance	Yes		10.1 ± 3.6	**0.020**	10.1 ± 3.6	**0.020**	10.2 ± 3.4	**0.001**	10.2 ± 4.3	**0.001**
	No		9.2 ± 4.1		9.2 ± 4.1		8.5 ± 4.6		8.5 ± 4.7	
Habitat	Urban		10.1 ± 3.7	0.700	10.1 ± 3.7	0.700	10.1 ± 3.6	0.100	10.1 ± 3.6	0.100
	Rural		9.8 ± 3.5		9.8 ± 3.5		9.8 ± 3.8		9.6 ± 3.8	
Education	Illiterate		9.2 ± 4.3	**0.030**	13.2 ± 3.5	**0.010**	9.0 ± 4.3	**0.010**	14.0 ± 5.5	**0.030**
	Under diploma		9.9 ± 3.7		14.1 ± 4.7		10.1 ± 3.6		15.3 ± 4.5	
	Diploma and higher		10.9 ± 2.3		15.5 ± 2.9		10.8 ± 2.8		16.0 ± 3.5	
Having any prior problem in the breast	Yes	Visit a doctor	11.8 ± 0.9	**0.001**	17.1 ± 1.6	**0.001**	11.6 ± 1.8	**0.001**	17.1 ± 2.5	**0.001**
		Failure to visit a doctor	10.6 ± 1.5		12 ± 3.7		9.1 ± 3.8		13.4 ± 3.1	
	No	None	9.9 ± 3.7		13.7 ± 4.9		9.7 ± 3.9		14.9 ± 4.9	
Types of breast problem	Breast pain		11.6 ± 0.9	0.200	15.9 ± 3.1	**0.001**	10.8 ± 2.9	0.070	15.9 ± 3.7	**0.003**
	Benign breast diseases		11.9 ± 0.4		17.6 ± 1.1		12.0 ± 0.1		17.7 ± 0.8	
	Discharge, infection, and abscess		11.4 ± 2.1		13.8 ± 4.8		11.5 ± 1.7		14.9 ± 4.8	
Breast cancer in first degree relatives	Yes		11.2 ± 2.8	**0.020**	16.7 ± 1.8	**0.050**	11.7 ± 1.4	**0.001**	16.4 ± 2.1	**0.050**
	No		9.9 ± 3.7		14.1 ± 4.7		9.9 ± 3.7		15.1 ± 4.7	
History of infertility	Yes		9.3 ± 4.2	0.070	13.6 ± 5.7	**0.030**	9.3 ± 4.2	**0.010**	14.9 ± 5.1	**0.030**
	No		10.1 ± 3.6		14.3 ± 4.5		10.1 ± 3.6		15.2 ± 4.6	
History of stillbirth	Yes		9.4 ± 4.1	0.200	14.2 ± 5.1	0.400	8.7 ± 4.7	**0.007**	14.2 ± 4.4	0.400
	No		10.1 ± 3.6		14.2 ± 4.7		10.1 ± 3.7		15.2 ± 4.5	
Number of abortions	Once		10.6 ± 3.1	0.100	14.1 ± 4.8	0.701	9.9 ± 3.6	0.500	15.2 ± 4.5	0.700
	Twice		9.6 ± 4.3		14.7 ± 4.3		9.1 ± 4.4		14.6 ± 4.9	
	More than twice		11.6 ± 1.1		14.7 ± 5.2		11.1 ± 2.6		16.1 ± 2.5	
History of pregnancy	Yes		10.1 ± 3.5	**0.003**	14.4 ± 4.5	**0.007**	9.9 ± 3.7	0.800	15.3 ± 4.5	0.007
	No		7.9 ± 4.7		11.7 ± 6.3		9.6 ± 4.1		13.9 ± 5.9	
History of mammography	Yes		11.9 ± 1.1	**0.001**	17.4 ± 1.7	**0.001**	11.1 ± 2.9	**0.001**	16.7 ± 3.4	**0.001**
	No		9.8 ± 4.1		12.6 ± 4.9		9.4 ± 3.9		14.4 ± 4.9	
Reason for mammography	Pain or mass in the breast		11.3 ± 2.8	0.400	16.3 ± 4.4	**0.010**	10.7 ± 3.7	0.500	15.9 ± 5.1	0.300
	Without breast problems and with the doctor's advice		11.9 ± 0.3		17.4 ± 1.3		11.4 ± 1.9		17.2 ± 1.8	
	Without breast problems and with the advice of others		11.9 ± 0.6		17.1 ± 1.1		10.3 ± 3.5		15.9 ± 4.1	

*Note:* Statistically significant *p*‐values are highlighted in bold.

Multivariate regression according to predictive factors associated with perceived benefits and barriers for BSE and mammography is presented in Table 4.

**TABLE 4:** Multivariate regression according to predictive factors associated with perceived benefits and barriers for BSE and mammography.

Predictive variable	Mammography						BSE					
	Benefits			Barriers			Benefits			Barriers		
	Reg.	SE	*p*	Reg.	SE	*p*	Reg.	SE	*p*	Reg.	SE	*p*
Age (35–45/45–55/55–65/≥ 65 years)	−0.069	0.019	**< 0.001**	−0.079	0.023	**< 0.001**	−0.038	−0.020	0.064	−0/058	0.025	**0.022**
Marital status (single/married/widow/divorced)	−0.0561	0.481	0.245	−0.901	0.581	0.121	−0.296	0.513	0.564	−0.529	0.644	0.412
Occupation status (house wife/employed)	−1.398	0.676	**0.039**	−1.772	0.815	**0.030**	−1.041	0.720	0.149	−1.056	0.905	0.224
Insurance status (yes/no)	0.430	0.425	0.312	0.218	0.512	0.670	1.345	0.452	**0.003**	1.314	0.568	**0.021**
Education level (illiterate/underdiploma/diploma and higher)	0.046	0.042	0.268	0.096	0.050	0.058	0.076	0.045	0.088	0.076	0.056	0.176
Visiting a doctor because of any Problem in the breast/no prior history of any breast discomfors	0.593	0.603	0.326	1.127	0.727	0.122	1.782	0.643	0.006	1.827	0.807	**0.024**
Types of breast problem (pain/benign breast disease/discharge, infection, and abscess)	1.014	0.699	0.147	0.063	0.834	0.940	0.028	0.744	0.971	−0.315	0.935	0.737
Breast cancer in first degree relatives (yes/no)	0.490	0.800	0.40	1.395	0.965	0.149	1.324	0.853	0.121	0.707	1.071	0.510
History of mammography (yes/no)	2.614	0.372	**< 0.001**	4.430	0.449	**< 0.001**	1.075	0.397	**0.007**	1.877	0.499	**0.000**
Reason for mammography (pain or mass in the breast/without breast problems and with the doctor's advice/without breast problems and with the advice of others)	−1.217	0.776	0.102	−1.50	0.936	0.0698	1.473	0.827	0.075	−1.835	1.039	0.078

*Note:* Perceived benefits of BSE was significantly associated with: Having insurance (*p* = 0.003), visiting a doctor because of a problem or discomfort in the breast (*p* = 0.006), and prior history of mammography (*p* = 0.007). Perceived barriers of BSE was significantly associated with: Age between 35 and 45 years (*p* = 0.022), having insurance (*p* = 0.021), visiting a doctor because of a problem or discomfort in the breast (*p* = 0.024), and prior history of mammography (*p* = 0.007). Perceived benefits of breast mammography was significantly associated with: Age between 35 and 45 years (*p* < 0.001), being house wife (*p* = 0.039), prior history of mammography (*p* = 0.000), and prior history of infertility (*p* = 0.036) and its perceived barriers was significantly associated with: age between 35 and 45 years (*p* < 0.001), Being house wife (*p* = 0.030), prior history of mammography (0.000), and prior history of infertility (*p* = 0.034). Statistically significant *p*‐values are highlighted in bold.

## 4 Discussion

In order to improve the prognosis of patients with breast malignancies, the leading cause of cancer‐related death among women, early detection and screening behaviors are essential. Early detection is restricted by a variety of barriers, including organizational and health‐care system‐level factors, as well as specific patient‐level obstacles. Furthermore, these barriers vary between nations due to economic development, social, and cultural impacts [24, 25]. This study was conducted to fill a critical gap in region‐specific evidence on perceived breast cancer screening barriers and benefits in Guilan, northern Iran, where no prior cohort‐based evaluations using the Champion Health Belief Model had been reported. By investigating women's knowledge barriers and benefits, we aimed to generate actionable insights for culturally tailored interventions.

Of the study participants, approximately 35% (*n* = 167) of them had a prior mammogram. According to an Iranian scoping review in consistency with our finding, the percentage of Iranian women who performed mammography, based on published data between 2005 and 2020, was 1.3%–45% [26]. Furthermore, a systematic review and meta‐analysis reviewing Persian publications from two Iranian databases involving the years 2001 to 2010 revealed that the mammogram test was conducted by 3%–26% of Iranian women [27]. In Bangladesh, as another low‐ and middle‐income country (LMIC), mammography is not optimally used due to limited resources [4]. It is quite different in the case of developed nations; based on the United States cancer control metrics, 75.9% (95% confidence interval [95% CI]: 74.7%–77.1%) of females aged 50–74 years received mammography within the past 2 years, without considering race/ethnicity [28]. Among the causes for this disparity could be the lack of a national commitment to a defined breast cancer screening education and program, as well as inequalities in economic status, educational level, and cultural settings [26]. Further national‐level measurements on this topic are needed to cover the mentioned gaps and develop a comprehensive guidelines in this regard.

Our results indicate that a substantial proportion of participants recognized the benefits of BSE and mammography, including early detection of breast lumps, reduced breast cancer mortality risk, and decreased anxiety, consistent with findings from previous studies [19, 29]. The predominant barrier for BSE identified in our study, “I don't feel I can do breast examination correctly,” reflects a significant lack of confidence and knowledge among women regarding BSE. This finding is consistent with numerous studies worldwide, highlighting inadequate skills and uncertainty about proper BSE techniques as major obstacles to regular practice. For instance, a study by Ewaid et al. in Iraq found that only 53% of studied women knew how to perform BSE, leading to a low practice rate of 24% [30]. Similarly, another study among female Jordanian students indicated that only 34.9% of those aware of breast cancer had knowledge about BSE, with actual practice reported by only 21.4% of them, suggesting a lack of understanding or awareness about the screening process among participants. A study by Akhtari‐Zavare et al. in Hamedan, Iran, stated that participants' knowledge about breast cancer and BSE is inadequate, with BSE practice significantly associated with breast cancer awareness [31]. These findings confirm the need for educational programs to improve BSE practice. In this regard, results from Zahedan, Iran, showed that the rate of BSE before and after a training program increased from 19% to 60.2% [32]. Another study by Beydağ and Yürügen among midwifery students in Turkey demonstrated that only around 40% of the participants had information about BSE, and only 25% practiced it. Following a BSE educational program, the level of knowledge increased significantly from 43.2 ± 10.6 to 68.4 ± 10.5 [33]. Regarding barriers of mammography, about 25% of the participants mentioned that “I don't know how to go about getting a mammogram,” underscoring the need for national and regional educational programs to promote breast cancer screening awareness and practice.

In this study, factors such as being younger (age between 35 and 45 years), having insurance, higher education levels, having any prior breast problems, a positive history of mammography, and a family history of breast cancer in first‐degree relatives were associated with better perception of the benefits and barriers of both BSE and mammography. As it was discussed earlier, educational obstacles are one of the most significant especially when it comes to self or clinical examinations, which are considered more available, low‐cost, and low‐technical need screening procedures in Iran as a developing nation. Consistent with our findings, a study conducted on females in Bangladesh revealed a significant correlation between the level of their education and their comprehension of breast cancer signs and symptoms, risk factors, as well as therapy possibilities. This suggests that a higher level of education is linked to greater awareness of the disease [34]. In addition to education, those with a familial background of breast cancer among their first‐degree family members exhibited notably elevated levels of perceived barriers and benefits associated with breast cancer screening. A parallel correlation was also seen in Saudi Arabia and Bangladesh regarding familial breast cancer history and awareness of disease screening [34, 35]. The current study has demonstrated that having insurance coverage, that provides free or reduced‐cost vouchers for medical services, such as clinical examinations or imaging procedures, has a major beneficial effect on the perception of barriers and benefits associated with breast cancer screening. This finding aligns with the outcomes of previous studies conducted in Japan and China and highlights the role of financial accessibility in screening uptake. Policymakers and healthcare administrators should consider subsidizing screening programs and improving physician‐driven referrals, particularly for uninsured women [36, 37].

In overall and as recommendations based on the study's findings, several targeted and culturally sensitive interventions can be implemented to improve both mammography uptake and BSE practices. First, educational programs should be designed to reach women who are most at risk, particularly older women and those with limited formal education and health insurance. These programs should focus not only on increasing awareness but also on empowering women with practical skills and confidence to perform BSE correctly. Community‐based workshops and visual materials can play a key role in overcoming fear, stigma, and misconceptions around breast cancer. Second, to reduce financial and access barriers, collaborations with insurance organizations and public health agencies are essential to offer free or low‐cost mammography, possibly through mobile screening units that can serve remote or underserved communities. These strategies not only respond directly to the barriers identified in this study but also affirm the autonomy of women by making screening services more accessible, understandable, and trustworthy. Investing in the above interventions can lead to earlier detection, better outcomes, and a stronger culture of preventive health.

It is important to take into account the limitations of this study. First, given that this study utilized a cross‐sectional design, it is possible that the analysis of any causal association may have been limited. Second, the use of self‐reported data to assess participants' perceptions and behaviors regarding breast cancer screening may introduce bias. Participants may overreport socially desirable behaviors, such as having undergone mammography, or underreport perceived barriers due to stigma, recall errors, or misunderstanding of the questions. Although we used a validated instrument (the Champion Health Belief Model Scale), self‐reporting inherently carries risks of subjective inaccuracies. Third, the selection of participants from the PERSIAN Guilan Cohort Study may limit generalizability. Women who are already part of this cohort may have higher health awareness or better access to healthcare facilities compared to the general population, which could have impacts on our findings. Additionally, those who declined participation may differ systematically in their screening behaviors and beliefs, introducing non‐response bias. Future longitudinal and interventional studies or qualitative investigations could provide deeper insight into evolving perceptions and actual screening behaviors over time.

## 5 Conclusion

According to the barriers and benefits identified in this study, it is possible and also necessary to plan for breast cancer screening with organized regional educational plans. It is recommended to focus more on attracting older women to perform screening programs. It is also necessary to encourage doctors to refer women for mammography and support insurance organizations to provide screening services at a lower cost.

## Author Contributions

F.J., S.Z., and F.M.‐G. participated in the research design. F.F., F.S., and F.J. participated in writing the first draft. F.F., F.S., S.Z., and F.H. participated in the performance of the research and analytic tools. Z.A.‐R. participated in data analysis. All authors reviewed and confirmed the final manuscript.

## Funding

The authors have nothing to report.

## Ethics Statement

Prior to their participation in the research, all patients provided informed consent for inclusion. The research was carried out in adherence to the principles outlined in the Declaration of Helsinki, and the research protocol received approval from the Ethics Committee of Guilan University of Medical Sciences (IR.GUMS.REC.1397.363).

## Consent

The authors have nothing to report.

## Conflicts of Interest

The authors declare no conflicts of interest.

## Data Availability

The data that support the findings of this study are available on request from the corresponding author. The data are not publicly available due to privacy or ethical restrictions.
